# Supplementary material for: Conductance Mechanisms of Rapidly Desensitizing Cation Channelrhodopsins from Cryptophyte Algae
Source: mBio. 2020 Apr 21;11(2):e00657-20. doi: 10.1128/mBio.00657-20 (PMC7175095; doi:10.1128/mBio.00657-20)
Supplement: FIG S1 [file mBio.00657-20-sf001.pdf]

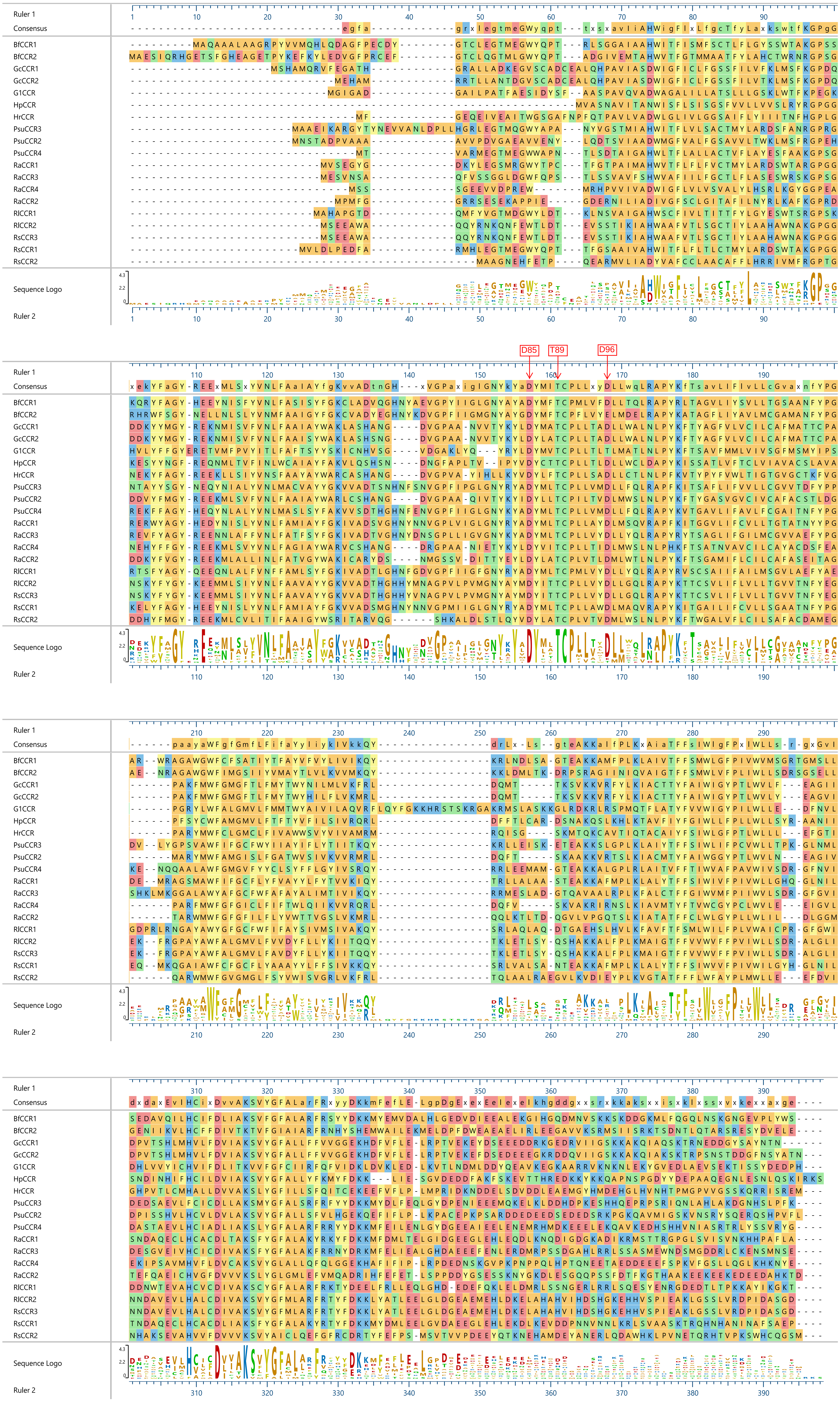

Ruler 1
Consensus
BfCCR1
BfCCR2
GcCCR1
GcCCR2
G1CCR
HpCCR
HrCCR
PsuCCR3
PsuCCR2
PsuCCR4
RaCCR1
RaCCR3
RaCCR4
RaCCR2
RlCCR1
RlCCR2
RsCCR3
RsCCR1
RsCCR2
Sequence Logo
Ruler 2
D85
T89
D96
