# Supplementary figures and images for: Conductance Mechanisms of Rapidly Desensitizing Cation Channelrhodopsins from Cryptophyte Algae
Source: mBio. 2020 Apr 21;11(2):e00657-20. doi: 10.1128/mBio.00657-20 (PMC7175095; doi:10.1128/mBio.00657-20)

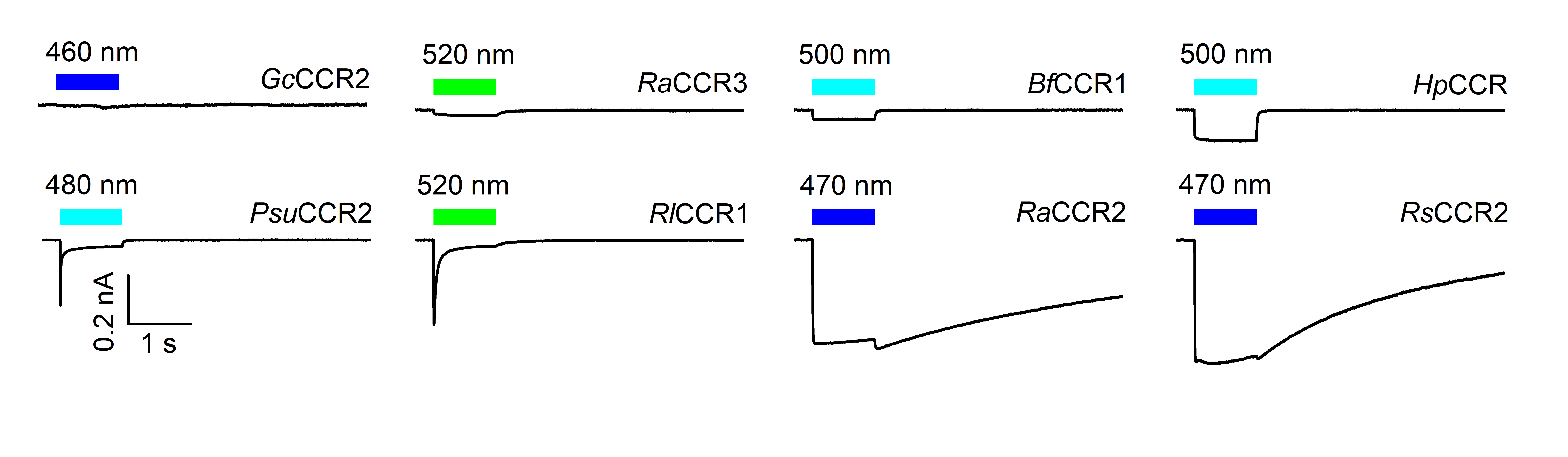

Supplement: FIG S3 [file mBio.00657-20-sf003.tif]

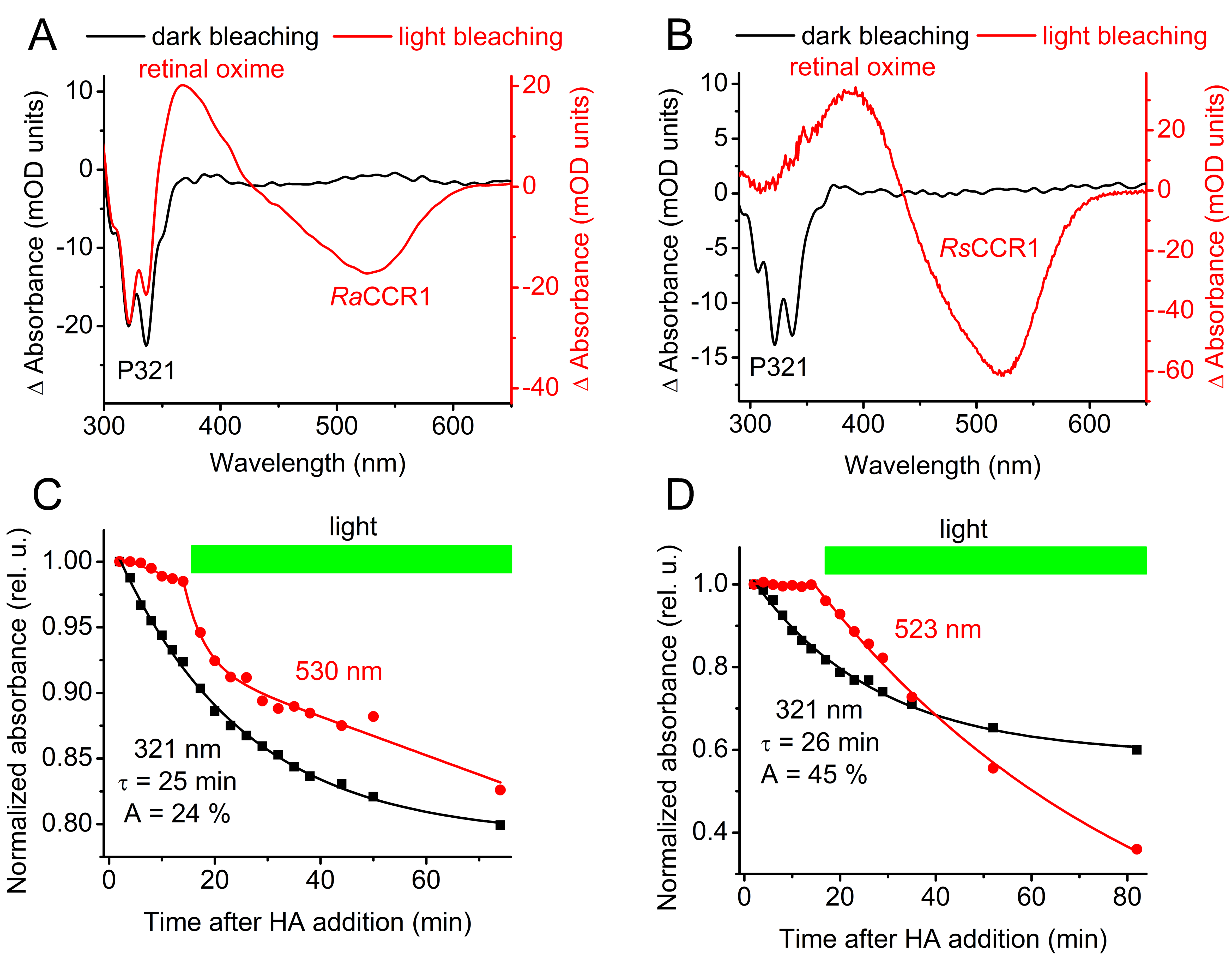

Supplement: FIG S5 [file mBio.00657-20-sf005.tif]

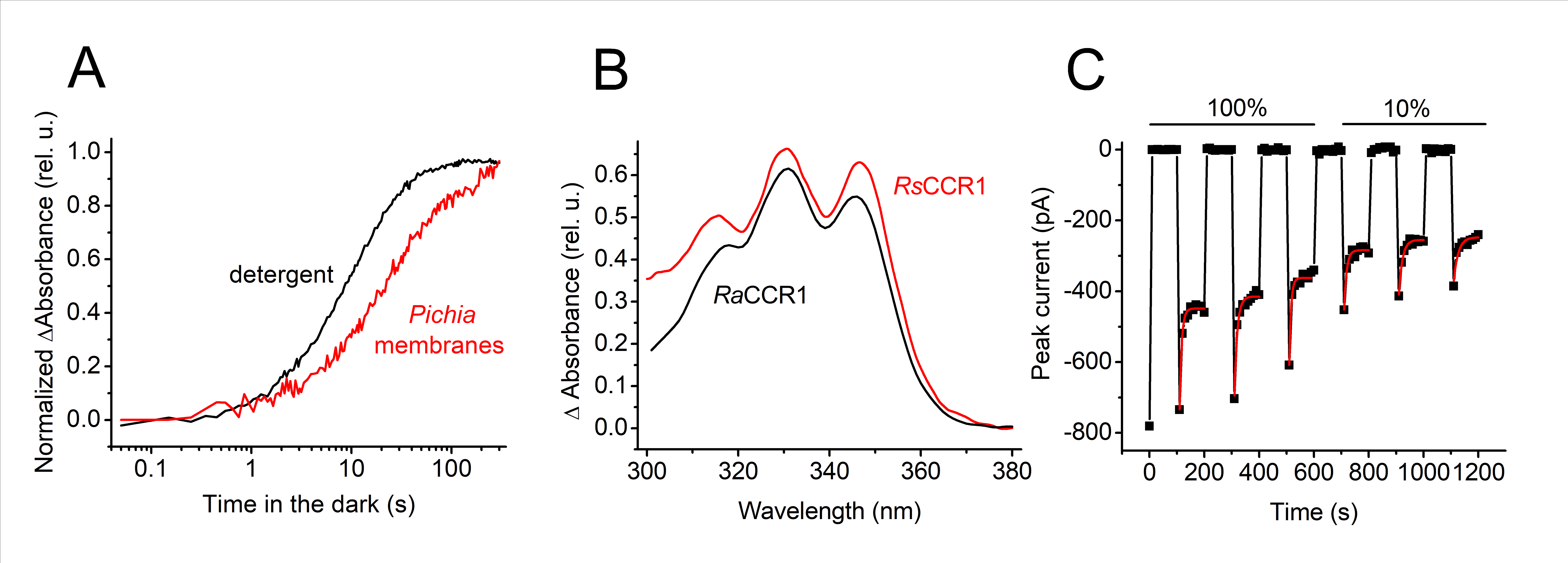

Supplement: FIG S6 [file mBio.00657-20-sf006.tif]

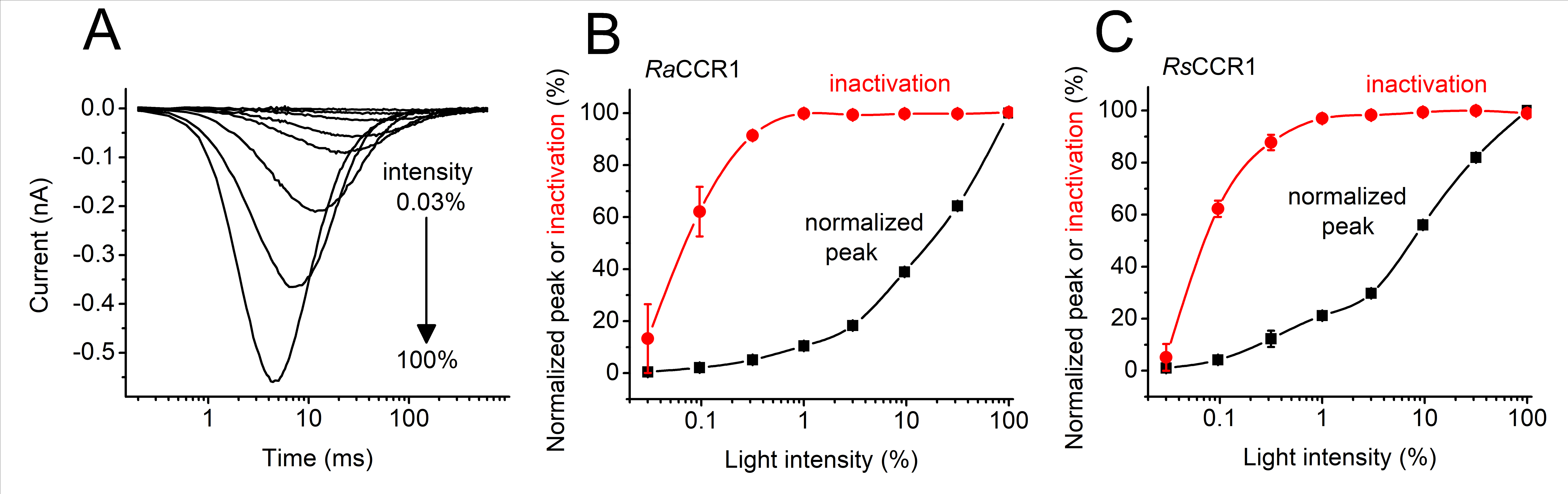

Supplement: FIG S7 [file mBio.00657-20-sf007.tif]

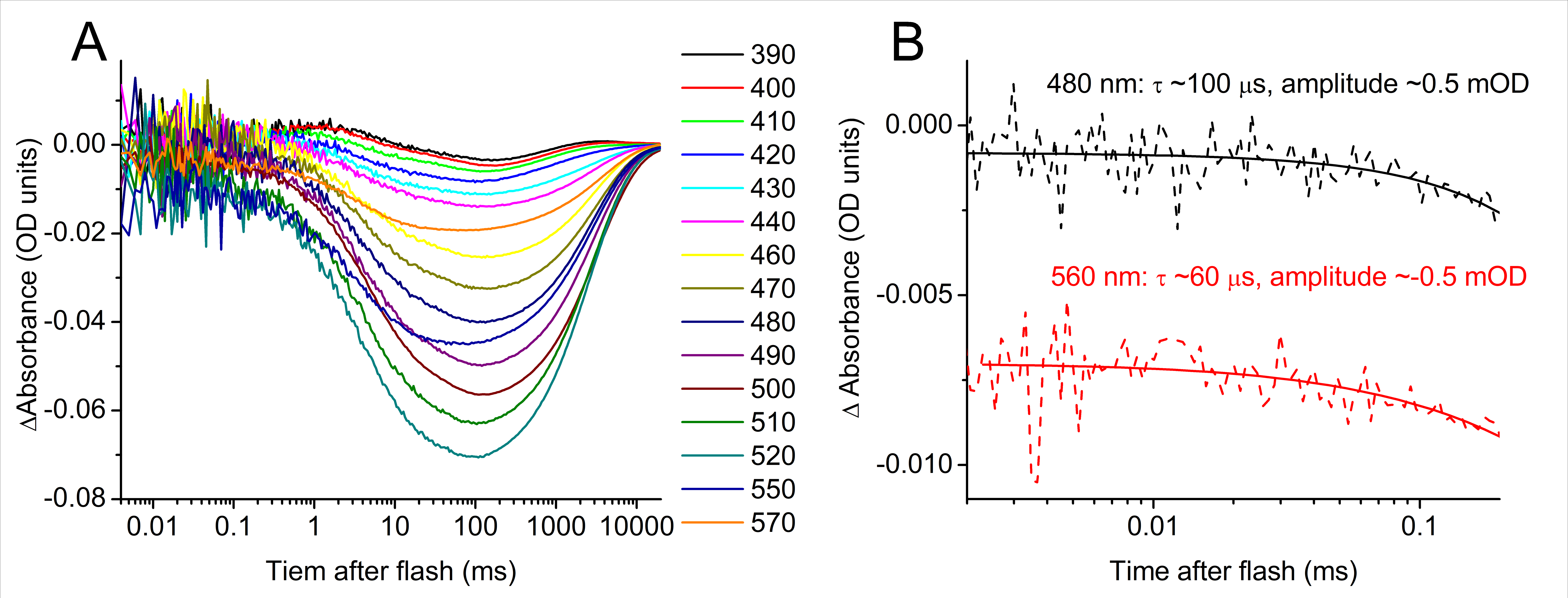

Supplement: FIG S8 [file mBio.00657-20-sf008.tif]

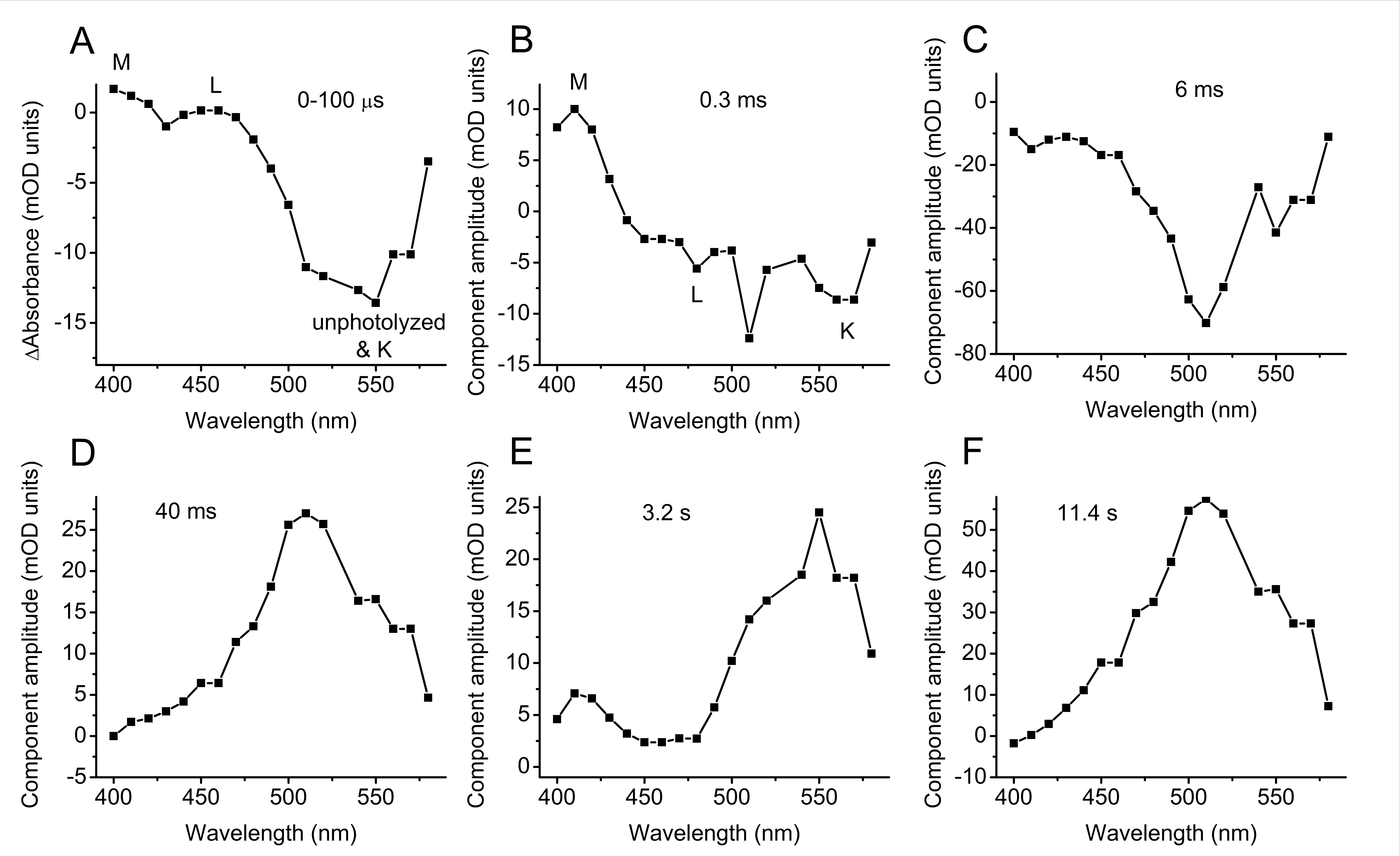

Supplement: FIG S9 [file mBio.00657-20-sf009.tif]

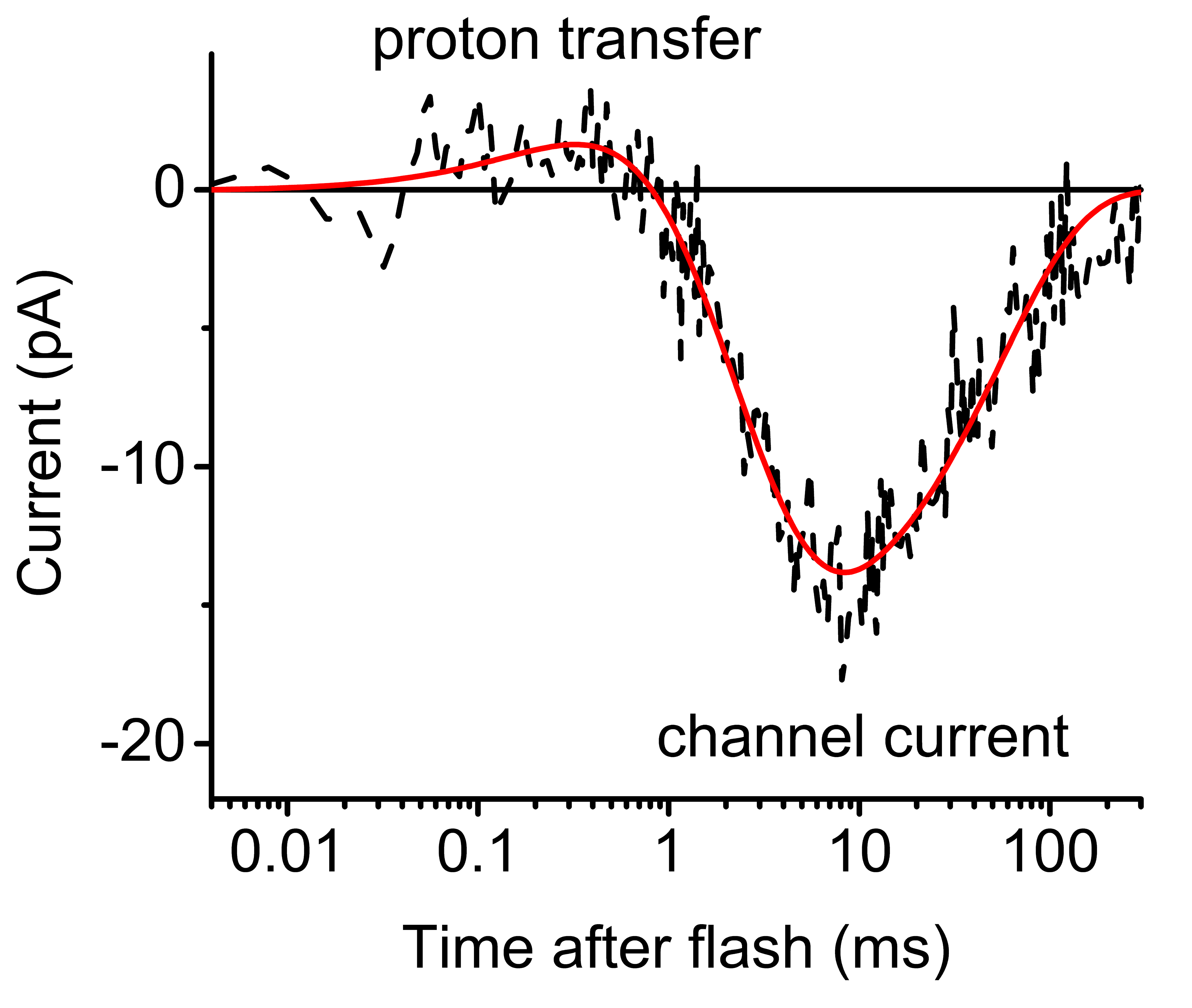

Supplement: FIG S10 [file mBio.00657-20-sf010.tif]
